# Cytokine therapy‐mediated neuroprotection in a Friedreich's ataxia mouse model

**DOI:** 10.1002/ana.24846

**Published:** 2017-02-23

**Authors:** Kevin C. Kemp, Nadia Cerminara, Kelly Hares, Juliana Redondo, Amelia J. Cook, Harry R. Haynes, Bronwen R. Burton, Mark Pook, Richard Apps, Neil J. Scolding, Alastair Wilkins

**Affiliations:** ^1^ Multiple Sclerosis and Stem Cell Group, School of Clinical SciencesUniversity of BristolBristolUnited Kingdom; ^2^ Sensory and Motor Systems Group, School of Physiology, Pharmacology and NeuroscienceUniversity of BristolBristolUnited Kingdom; ^3^ Brain Tumour Research Group, School of Clinical SciencesUniversity of BristolBristolUnited Kingdom; ^4^ Infection and Immunity, School of Cellular and Molecular MedicineUniversity of BristolBristolUnited Kingdom; ^5^ Synthetic Biology Theme, Institute of Environment, Health & Societies, Biosciences, Dept. of Life Sciences, College of Health & Life SciencesBrunel University LondonLondonUnited Kingdom

## Abstract

**Objectives:**

Friedreich's ataxia is a devastating neurological disease currently lacking any proven treatment. We studied the neuroprotective effects of the cytokines, granulocyte‐colony stimulating factor (G‐CSF) and stem cell factor (SCF) in a humanized murine model of Friedreich's ataxia.

**Methods:**

Mice received monthly subcutaneous infusions of cytokines while also being assessed at monthly time points using an extensive range of behavioral motor performance tests. After 6 months of treatment, neurophysiological evaluation of both sensory and motor nerve conduction was performed. Subsequently, mice were sacrificed for messenger RNA, protein, and histological analysis of the dorsal root ganglia, spinal cord, and cerebellum.

**Results:**

Cytokine administration resulted in significant reversal of biochemical, neuropathological, neurophysiological, and behavioural deficits associated with Friedreich's ataxia. Both G‐CSF and SCF had pronounced effects on frataxin levels (the primary molecular defect in the pathogenesis of the disease) and a regulators of frataxin expression. Sustained improvements in motor coordination and locomotor activity were observed, even after onset of neurological symptoms. Treatment also restored the duration of sensory nerve compound potentials. Improvements in peripheral nerve conduction positively correlated with cytokine‐induced increases in frataxin expression, providing a link between increases in frataxin and neurophysiological function. Abrogation of disease‐related pathology was also evident, with reductions in inflammation/gliosis and increased neural stem cell numbers in areas of tissue injury.

**Interpretation:**

These experiments show that cytokines already clinically used in other conditions offer the prospect of a novel, rapidly translatable, disease‐modifying, and neuroprotective treatment for Friedreich's ataxia. Ann Neurol 2017;81:212–226

Friedreich's ataxia, the commonest autosomal‐recessive ataxic condition, is characterized neuropathologically by degeneration of large sensory neurons, the spinal cord, and the deep cerebellar nuclei.^1^ Friedreich's ataxia patients inevitably acquire neurological disability, with progressive ataxia, dysarthria, neuropathy, and pyramidal weakness, as well as cardiac and endocrine disease.^2^ In most cases, Friedreich's ataxia is caused by homozygous GAA.TTC trinucleotide repeat expansion within intron 1 of the *FXN* gene,^3^ causing transcriptional repression of frataxin.^4^ Frataxin is an essential mitochondrial protein, loss of which causes disrupted respiratory chain activity, impaired cellular iron homeostasis, and oxidative stress, leading to cell death in affected tissues.^5^, ^6^, ^7^

Currently, there are no treatments that can protect nerves, promote nervous system regeneration, or slow disease progression.^8^ However, specific cytokines and growth factors may show therapeutic potential in mediating nervous system injury and repair. Originally used clinically for stimulating and mobilising specific subsets of bone marrow stem and progenitor cell populations preceding a peripheral blood stem cell harvest, the agents, granulocyte‐colony stimulating factor (G‐CSF) and stem cell factor (SCF),^9^, ^10^ may also exhibit direct protective and reparative effects within diseased tissues and, in combination, show significant biological and clinical synergy with each other.^11^ Therapeutically, G‐CSF and SCF are known to cross the blood–brain barrier,^12^ exert antiapoptotic effects on glial and/or neural cells,^13^, ^14^ stimulate neurogenesis and neurite outgrowth,^15^, ^16^ and promote migration of endogenous neural progenitor cells.^17^ Furthermore, these agents promote accelerated recovery in several animal models of neurological disease^18^ and are already in human clinical trials for other neurological disorders.^19^ Here, we study the therapeutic potential of the repeated subcutaneous administration of both G‐CSF and SCF in a humanized murine Friedreich's ataxia model. Using recognized cytokine regimens for the mobilization of murine peripheral blood hematopoietic stem cells (HSCs), we demonstrate for the first time, major benefits in clinical, biochemical, neurophysiological, and pathological parameters associated with the disease.

## Materials and Methods

### Experimental Design

**Figure 1:**
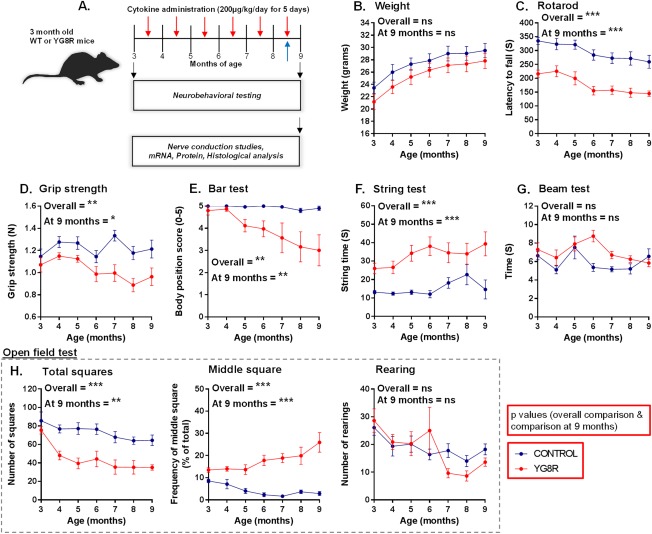
Neurological deficits in YG8R mice that carry a human genomic *FXN* transgene containing expanded GAA repeats of 82 to 190 units within intron 1 of *FXN*. (A) Experimental protocol using wild‐type controls (WT) and YG8R mice to investigate the effects of cytokine administration on disease phenotype. Mice received monthly infusions of cytokines (red arrows) while also being assessed at monthly time points using an extensive range of behavioral performance tests. Bromodeoxyuridine was also administered during the last round of cytokine treatment (blue arrow). At 9 months of age, neurophysiological evaluation of both sensory and motor nerve conduction was performed. Subsequently, mice were sacrificed for mRNA, protein, and histological analysis. Comparisons between WT‐control and untreated YG8R mice: longitudinal results for (B) weight; (C–G) motor performance; and (H) locomotor performance (open field test) in mice from 3 to 9 months of age. Repeated measures two‐way analysis of variance was applied for all behavioral studies. *p < 0.05;**p < 0.01; ***p < 0.001; values represent means ± standard error of the mean. For all tests, n = 10 (5 female and 5 male) per genotype. mRNA = messenger RNA; ns = not significant.

The objectives of the study were to investigate, in a mouse model of Friedreich's ataxia (YG8R mice), the therapeutic effects of cytokine administration (G‐CSF/SCF) on disease phenotype. Experimental protocols are described in Fig 1A. Investigators were blinded to treatment group for behavioral studies.

### Animals

All animal experiments were performed in accord with the UK Animals (Scientific Procedures) Act 1986 and approved by the University of Bristol Animal Welfare and Ethical Review Body. *Fxn* ^*tm1Mkn*^ Tg(FXN)YG8Pook/J (YG8R) transgenic mice, which carry a human genomic *FXN* transgene (on a murine frataxin null background) containing expanded GAA repeats of 82 to 190 units within intron 1 of *FXN*, were used. Ataxic (strain no. *Fxn* ^*tm1Mkn*^ Tg(FXN)YG8Pook/J; stock no. 008398) were purchased from The Jackson Laboratory (Bar Harbor, ME). Control C57BL/6 VAF/Elite mice were provided by Charles River Laboratories (Margate, UK). All mice were housed in a pathogen‐free facility, with free access to food and water.

### Treatment

Cytokine doses were based on standard regimens for G‐CSF mobilization of peripheral blood HSCs. In humans, 5 to 10 µg kg/day of recombinant G‐CSF is administered for 4 to 7 days (British National Formulary; BNF^20^). Mobilizing doses of G‐CSF for murine HSCs is considerably higher than for humans to achieve similar levels of mobilization,^21^ commonly 100 to 300 µg/kg/day is injected for 5 or more days.^22^, ^23^, ^24^ The rationale to use cytokines G‐CSF and SCF at a single fixed dosage (200 µg/kg) was to allow comparisons to be made between the effectiveness of the two cytokines when used alone.

Mice received subcutaneous injection of murine G‐CSF and/or SCF (both 200 µg/kg; PeproTech, Rocky Hill, NJ) in phosphate‐buffered saline (PBS) once‐daily for 5 consecutive days. Treatments were repeated every 4 weeks. PBS alone was administered as a vehicle control, during the same period. Bromodeoxyuridine (BrdU; 50mg/kg; Sigma‐Aldrich, St. Louis, MO) in PBS was also administered intraperitoneally once‐daily for 5 consecutive days (directly following growth factor infusion) during the last treatment round.

### Peripheral Blood Mononuclear Cell Counts

One hundred microliters of peripheral blood (PB) was harvested from the tail vein and suspended in PBS (pH 7.4)/ethylenediaminetetraacetic acid (2mg/ml). Red cells were removed using red cell lysis buffer, the remaining nucleated cell population resuspended in PBS/3% fetal bovine serum and counted using a hemocytometer.

### Neurobehavioral Testing

#### Body Weight

Mouse body weights were recorded once‐monthly between 3 and 9 months of age using a digital scale.

#### Rotarod

A digital rotarod, rod diameter 30mm, accelerating from 4 to 40rpm over 400 seconds was used (Ugo Basile 47600; Ugo Basile S.R.L., Gemonio, Italy). Mice were allowed to stand on the slowly rotating (4rpm) rod for 30 seconds before acceleration. Each month, mice were first trained on the rotarod using three unrecorded trials with 15‐minute intertrial intervals. Six hours later, mice received four recorded trials with 15‐minute intertrial intervals. All trials ended when the mouse fell off the rotarod or after a maximum 400 seconds had elapsed. The time that each mouse maintained its balance on the rotating rod was measured as the latency to fall.

#### Grip Strength

A 2.5‐Newton meter was used to assess forelimb grip strength. Mice were held by the base of the tail and allowed to grasp a metal bar attached to the meter. The peak force with which mice pulled the bar horizontally was measured in three trials with a rest period of 1 minute between each trial.

#### Bar Hang Test

Mice were placed with their forepaws grasping the middle of a 200‐mm horizontal wooden bar (4mm thick), secured to a vertical post, elevated 300mm from a flat surface. The ability to grip the bar was scored as following: 0 unable to remain on bar; 1, hangs by both forepaws; 2, attempts to climb onto bar; 3, both forepaws and one or both hindpaws around bar; 4, four paws and tail around bar, with lateral movement; and 5, escape. The score was measured in three trials with a rest period of 5 minutes between each trial.

#### String Test

Mice were placed with their forepaws grasping the middle of a taut 1‐m horizontal rope (4mm thick), suspended between two vertical supports, elevated 300mm from a flat surface. The time taken for each mouse to reach either end of the rope was measured. Trials ended when the mouse fell off the rope or after a maximum 60 seconds had elapsed. Latency was measured in three trials with a rest period of 5 minutes between each trial.

#### Beam‐Walk Test

The test was carried out using a wooden beam 900mm long, with an external diameter of 10mm. Within a darkened room, the beam was placed between two platforms sloping up from 300 to 600mm above the bench surface with the lower end mounted on a narrow support with a 60‐W lamp, while a darkened safety platform was located at the other end of the beam, in which the mouse could escape. Performance on the beam was quantified by measuring the time it took the mouse to traverse the beam and enter the safety platform. Latency was measured with a rest period of 5 minutes between each trial.

#### Open Field

Within a darkened room, mice were placed into a 3 × 3 gridded (300 × 300mm size) clear Perspex box with 150‐mm‐high walls. A 60‐W lamp illuminated the arena. The total number of grid squares entered, the frequency of central square entered, and the total number of hindpaw rearings by each mouse over a period of 5 minutes was recorded. Scores were measured with a rest period of 30 minutes between each trial.

### Gait Analysis

**Figure 2:**
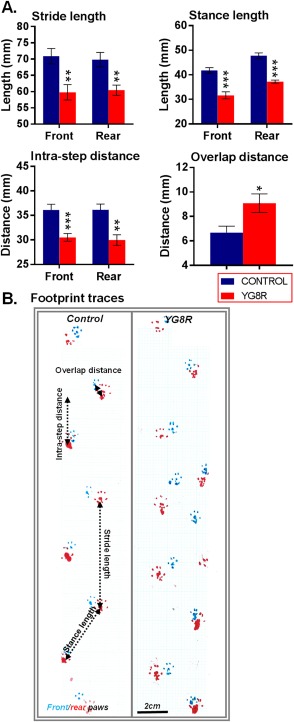
YG8R mice show gait abnormalities. Comparisons between WT‐control and untreated YG8R mice: (A) footprint (gait) analysis and (B) representative footprint traces in mice 9 months of age. The unpaired *t* test was applied for all analysis. *p < 0.05; **p < 0.01; ***p < 0.001; values represent means ± standard error of the mean. For all tests, n = 10 (5 female and 5 male) per genotype. WT = wild type.

To obtain footprints, hind‐ and forepaws of the mice were coated with red and green nontoxic food coloring, respectively. The animals were then allowed to walk along a 500‐mm‐long, 55‐mm‐wide runway with 100‐mm‐high walls (lit at the entrance with a 60‐W lamp), with white paper lining the floor, into a darkened enclosed escape box. All mice received both one training and trial run. A fresh sheet of paper was placed on the floor of the runway for each run. The footprint patterns of the hind‐ and forepaws were analyzed for four‐step parameters (all measured in millimeters): stride length; stance length; intra‐step distance; and overlap distance (shown in Fig 2).

### Neurophysiology

Animals were anesthetized with ketamine (100mg/kg; Vetalar; Boehringer Ingelheim Vetmedica Inc., St. Joseph, MO) and xylazine (10mg/kg; Rompun; Bayer Plc, Newbury, UK) intraperitoneally. Depth of anesthesia was regularly assessed by a paw pinch to monitor reflex muscle tone, and supplementary doses were administered as required. Core body temperature was maintained at 37°C by a heated blanket.

#### Tail Nerve Conduction Studies

Assessment of the conduction velocity of sensory and motor fibers was performed by stimulating the distal and proximal parts of the tail, respectively, and recording evoked responses.^25^, ^26^ For stimulation of the sensory nerves, two uninsulated needles with a separation of approximately 5mm were inserted at the tip of the tail with the cathode. The cathode was the most proximal of the needles to the base of the tail. Bipolar recordings were made using needle electrodes inserted approximately 20 (position A) and 40mm (position B) from the cathode. The reference electrode was inserted above the base of the tail. For stimulation of motor nerves, the same electrode positions were used except that the electrodes in the tip of the tail were used to record the responses and stimulation delivered at positions A and B.

Electrical stimulation consisted of constant current square pulses of 0.2ms duration delivered every 3 seconds. Recordings were made with a stimulus intensity ×3 threshold to evoke a response. Nerve compound potentials were amplified (×1,000), bandpass filtered (5Hz–5kHz) and digitized online using a Cambridge Electronic Design (CED, Cambridge, UK) Micro 1401 analog‐to‐digital converter and Spike2 software (CED). Latency of responses was taken from stimulus onset time to the beginning of the potential as well as to the peak. The distance between the two recording sites was measured with a calliper and the sensory and motor nerve conductions calculated by dividing the distance by the difference in latencies of the proximal and distal recording sites. For each animal analysis of sensory and motor conduction, velocities and nerve volley duration was based on an average of 10 trials.

### Tissue Processing

Mice were sacrificed and tissues for both proteomic and genomic analyses were dissected, immediately snap‐frozen in liquid nitrogen, and stored at –80 ºC until use. Frozen tissue was then thawed and homogenized on ice by use of the PARIS kit (Ambion, Cambridge, UK) and a protease and phosphatase inhibitor cocktail (1:100; Fisher Scientific UK Ltd., Loughborough, UK).

For histological analyses, mice were anesthetized by intraperitoneal injection of Euthatal and perfused with PBS followed by 4% paraformaldehyde (PFA) in PBS. Brains and spinal cords were dissected and placed in 4% PFA in PBS for 24 hours at 4°C and subsequently embedded in paraffin for sectioning on a rotary microtome (Leica LM2135; Leica Microsystems, Buffalo Grove, IL) and mounting on glass slides.

### Gene Expression Analysis: Quantitative Polymerase Chain Reaction

Total RNA was extracted from tissue lysates on ice using the PARIS kit (Ambion), according to manufacturer's instructions, and treated with DNase I recombinant (Roche Diagnostics, Indianapolic, IN)/MgCl_2_ solution (Bioline, London, UK). All RNA samples were quantified using a Qubit Fluorometer and Quant‐iT RNA assay kit (Invitrogen, Paisley, UK), according to manufacturer instructions. RNA was reverse transcribed to produce complementary DNA (cDNA) using the High Capacity cDNA Kit (Applied Biosystems, Foster City, CA) and quantitative polymerase chain reaction (qPCR) performed using TaqMan Fast Advanced Master Mix (Applied Biosciences, Paisley) and the StepOnePlus Real‐Time PCR System (Applied Biosystems, UK) with primers for *Epas1* (Mm01236112_m1), *FXN* (Hs00175940_m1), *Srf* (Mm00491032_m1), *Tfap2a* (Mm00495574_m1), and *Trp53* (Mm01731290_g1; TaqMan MGB probe, FAM dye‐labeled; Applied Biosystems, UK). Relative gene expression (relative quantities [RQ] value) was calculated using the 2^‐ΔΔCt^ method with both Beta Actin (*Actb*; Mm00607939_s1) and NeuN (*Rbfox3*; Mm01248771_m1) used as housekeeping genes.

### Protein Analysis

The Qubit Fluorometer and Quant‐iT protein assay kit (Invitrogen, UK) was used to quantify the concentration of total protein within each tissue homogenate. Frataxin levels were measured using the Frataxin Human SimpleStep enzyme‐linked immunosorbent assay (ELISA) kit (Abcam), and enzyme activity of Aconitase was determined using the Aconitase assay kit (Cayman Chemical, Ann Arbor, MI).

For further quantitative protein analysis, immunodot blotting was carried out using the Bio‐Dot Microfiltration manifold system (Bio‐Rad Laboratories, Hercules, CA). Protein homogenates were transferred to the nitrocellulose membrane using gravity filtration, blocked using 5% bovine serum albumin, before incubation with the following primary antibodies: catalase (Abcam; ab16731 1:5000); glutathione peroxidase 1 (GPX1; 1:5,000; ab22604; Abcam); 4‐hydroxynonenal (4‐HNE; 1:6,000; ab48506; Abcam); nuclear factor E2‐related factor 2 (Nrf2; 1:2,000; sc‐722; Santa Cruz Biotechnology, Santa Cruz, CA); peroxisome proliferator‐activated receptor‐gamma coactivator 1 alpha (PGC‐1a; 1:2,000; sc‐13067; Santa Cruz Biotechnology); neuronal nuclear antigen (NeuN; 1:4,000; ab177487; Abcam); superoxide dismutase 1 (SOD1; 1:5,000; ab16831; Abcam); and superoxide dismutase 2 (SOD2; 1:20,000; ab16956; Abcam). Immunoreactivity was detected using horseradish peroxidase–conjugated goat antimouse immunoglobulin G (IgG; 1:5,000; ab6789; Abcam) or antirabbit IgG (1:3,000; ab6721; Abcam) secondary antibodies. Protein expression was visualized using a chemiluminescence EZ‐ECL kit in conjunction with a Bio‐Rad Universal III Bioplex imager. Densitometric analysis of protein expression was performed using Image Lab software (version 5.0; Bio‐Rad).

#### Immunohistochemistry and Imaging

Mounted tissue sections were deparaffinized, rehydrated, and washed with PBS. For antigen‐exposing pretreatment, sections were incubated with boiling 0.01M of sodium citrate buffer (pH 6.0). Immunohistochemical staining with 3,3’‐diaminobenzidine has been described previously.^27^ For immunofluorescent labeling, nonspecific binding was blocked with 10% normal goat serum diluted in PBS containing 0.1% Triton. Sections were incubated at 4°C overnight with primary antibodies to 4‐HNE (1:200; ab48506; Abcam), beta‐3 tubulin (1:250; ab78078; Abcam), BrdU (B2531; Sigma‐Aldrich), Calbindin‐D28K (1:500; C2724; Sigma‐Aldrich), glutamate decarboxylase (GAD; 1:1,000; ab11070; Abcam), glial fibrillary acidic protein (GFAP; 1:200; ab33922; Abcam), myelin basic protein (MBP; 1:100; MCA4095; AbD Serotec, Kidlington, UK), NeuN (ab177487 [1:500] and ab104224 [1:500]; Abcam), Nestin (1:200; 556309; BD Biosciences, San Jose, CA), OX42 (1:100; ab1211; Abcam), S100 (1:200; MAB079; Milipore, Billerica, MA), and S100 (1:200; Z0311; Dako, Carpinteria, CA). Sections were washed in PBS and incubated for 45 minutes in the dark with Alexa Fluor 488/555, goat antimouse (1:500), or Alexa Fluor 488/555, goat antirabbit (1:500; Invitrogen, UK) before being mounted in VECTASHIELD medium containing the nuclear dye, 4′,6‐diamidino‐2‐phenylindole (DAPI; H‐1200; Vector Laboratories). For BrdU labeling, sections were incubated at 37°C in 2N of HCl for 30 mins followed by 0.1% Trypsin for 20 minutes before blocking.

Confocal analysis was performed using either a Leica SP5‐AOBS confocal laser scanning microscope attached to a Leica DM I6000 inverted epifluorescence microscope (Leica Microsystems) or a Nikon C1 confocal microscope (Nikon, Tokyo, Japan) and EZ viewer software. All Z‐stack and three‐dimensional (3D) imaging was created using both Leica Application Suite Advanced Fluorescence software and Volocity 3D image software (PerkinElmer, Waltham, MA). For light imaging, images were acquired using an Olympus IX70 microscope (Olympus, Tokyo, Japan) coupled with Image‐Pro Plus software.

### Histological Staining

For histological assessment, tissues were sectioned, deparaffinated, rehydrated, and stained with hematoxylin and eosin (H&E; visualisation of dorsal root ganglia [DRG] vacuoles) or Luxol fast blue/cresyl violet (visualisation of myelin).

### Cell Quantification

At least four independent tissue samples from each group were included in the analyses. All cells were counted within randomly assigned set areas within the DRG, spinal cord, and cerebellar dentate nucleus. For spinal cord sections, representative samples of cervical, thoracic, and lumbar were all analyzed.

### DRG Vacuoles

Each section was scanned across the entire cross‐sectional area of the DRG for neuronal cell bodies containing either nuclear and/or cytoplasmic vacuoles. A minimum of 400 DRG neurons from each mouse was examined, allowing for the determination of the frequency of vacuolated cells.

#### Neuronal Cell Size

To quantify changes in neuronal size, the cross‐sectional diameter of a nucleated cell soma was measured in each cell using ImageJ software (National Institutes of Health [NHI], Bethesda, MD). Neurons were identified by either beta‐3 tubulin (cerebellar dentate nucleus) or NeuN/H&E (DRG).

### Statistical Analysis

The analysis was performed using GraphPad Prism software (GraphPad Software Inc., San Diego, CA. For all tests, values of *p* < 0.05 were considered statistically significant. Statistical tests were all two‐sided. At least five independent tissue samples or mice from each group were included in the analyses. Data between two groups were analyzed using either unpaired *t* tests or Mann–Whitney *U* tests. Statistical comparisons for over two groups were analyzed using either Friedman's test or one‐ or two‐way analysis of variance with post‐hoc testing between groups where appropriate (as indicated in figure legends). Where possible, data are represented as mean ± standard error of the mean, or for qPCR data geometric mean ± 95% confidence intervals are stated.

## Results

To investigate G‐CSF/SCF administration, we used 3‐month *Fxn* ^*tm1Mkn*^ Tg(FXN)YG8Pook/J (YG8R) transgenic mice, which carry a human genomic *FXN* transgene (on a murine frataxin null background) containing expanded GAA repeats within intron 1 of *FXN*. Mice are frataxin‐deficient and develop progressive neurodegeneration and cardiac pathology.^28^, ^29^ Because of the phenotypic similarity between C57BL/6 and Y47R mice (Y47R mice carry the human *FXN* transgene with normal‐sized GAA repeats), C57BL/6 mice were used as healthy controls.^29^ Before any therapeutic intervention, neurological deficits were already apparent in the YG8R mice compared to age‐matched wild‐type (WT) controls. These deficits in the YG8R mice became more prominent with increasing age (Figs 1C–H and Fig 2).

### G‐CSF and SCF Improve Both Motor and Locomotor Performance in YG8R Mice

**Figure 3:**
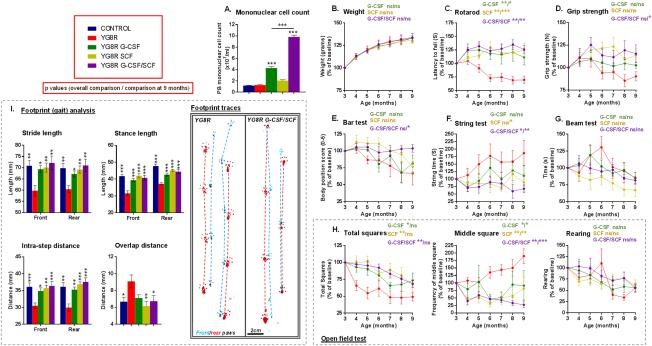
Treatment with G‐CSF and/or SCF improves both motor and locomotor performance in YG8R mice. (A) The peripheral blood MNC counts of WT control and YG8R mice subcutaneously injected with G‐CSF and/or SCF dissolved in PBS (200 μg/kg body weight daily for 5 consecutive days). Longitudinal results for (B) weight; (C–G) motor performance; (H) locomotor performance (open field test); and (I) gait analysis in YG8R mice treated with G‐CSF and/or SCF from 3 to 9 months of age. All statistical comparisons are versus YG8R mice using either the unpaired *t* test, one‐way or repeated measures two‐way analysis of variance, followed by Dunnett's multiple comparison test. *p < 0.05; **p < 0.01; ***p < 0.001; values represent means ± standard error of the mean. For all neurobehavioral tests, n = 10 (5 female and 5 male) per genotype. G‐CSF = granulocyte colony‐stimulating factor; MNC = mononuclear cell; ns = not significant; PBS = phosphate‐buffered saline; SCF = stem cell factor; WT = wild type.

YG8R transgenic mice were subcutaneously injected with PBS (untreated controls) or G‐CSF and/or SCF dissolved in PBS (daily for 5 consecutive days) monthly for 6 consecutive months (Fig 1A). For the duration of the study, no observable side effects in the mice were noted post‐G‐CSF and/or ‐SCF administration, weights remained consistent/normal (Fig 3B), and all mice (n = 50) completed the study. Combined G‐CSF/SCF increased peripheral blood mononuclear cell (MNC) counts approximately 8‐fold and was a more effective mobilizing regimen compared to administration of single agents (Fig 3A).

Motor coordination and locomotor activity in YG8R and control mice were assessed monthly. Significantly, after 6 months of G‐CSF and/or SCF administration, improvements were observed in the majority of motor coordination and locomotor activities tested (Fig 3C–I). Performances were independent of changes in body mass (Fig 3B).

### G‐CSF and SCF Increase Frataxin Messenger RNA and Protein Expression

**Figure 4:**
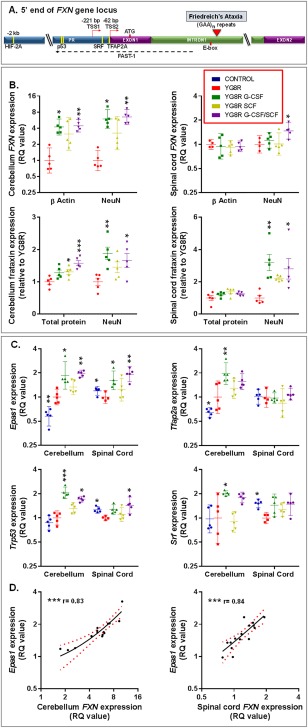
Both frataxin and regulatory factors implicated in controlling frataxin transcription are elevated in the cerebellum and spinal cord of YG8R mice treated with G‐CSF and/or SCF. (A) A schematic of the 5’ end of the human frataxin (*FXN*) gene showing approximate locations of the binding sites (yellow bars) for HIF‐2A, SRF, TFAP2A, and p53 in human or murine cells. The locations of the promotor (PR), Exon1, Exon2, and Intron1 regions are depicted. Different transcription start sites (TSS1 and TSS2) are shown upstream of Exon1, which holds the ATG translation start site. The directions of transcription for *FXN* (red arrows) and *FXN* antisense transcript (*FAST‐1*; dashed black arrow) are shown. The red triangle indicates the site of the trinucleotide GAA repeat expansion within intron 1 of *FXN* gene of patients with Friedreich's ataxia. The relative (B) mRNA and protein expression levels of frataxin within the cerebellum and spinal cord of YG8R mice (normalized to NeuN or β actin); (C) mRNA expression levels of transcription factors implicated in controlling frataxin expression *Epas1, Srf, Tfap2a*, and *Trp53* (normalized to NeuN). (D) Correlation and linear regression analysis of *FXN* and *Epas1* mRNA levels (normalized to NeuN) in the spinal cord and cerebellum of treated YG8R mice (lines of best fit and 95% confidence interval [CI] are depicted); r = Spearman's correlation coefficient. All statistical comparisons are versus YG8R mice. Comparisons between control and untreated YG8R mice were analyzed using unpaired *t* tests or Mann–Whitney *U* tests. For all other analyses, either one‐way analysis of variance followed by Dunnett's multiple comparison test or Kruskal‐Wallis followed by Dunn's multiple comparison test was applied. *p < 0.05; **p < 0.01; ***p < 0.001. For mRNA and protein expression, values represent the geometric means ± 95% CI and means ± standard error of the mean, respectively, relative to values in untreated YG8R mice. For all tests, n = 4 or 5 per genotype. mRNA = messenger RNA; NeuN = neuronal nuclear antigen; RQ = relative quantities.

Transcriptional repression of *FXN* is the primary molecular event in the pathogenesis of Friedreich's ataxia.^3^, ^4^ To explore potential neuroprotective mechanisms of G‐CSF and SCF, we measured frataxin messenger RNA (mRNA) levels in cerebellum and spinal cord of YG8R mice (aged 9 months) 24 hours postinjection of cytokines. *FXN* mRNA levels were significantly amplified following treatment, with G‐CSF or G‐CSF/SCF (cerebellum) and G‐CSF/SCF (spinal cord) having the most pronounced effects (Fig 4B). Notably, in all cases, increases in frataxin mRNA expression were more prominent using the neuronal‐specific marker, NeuN, housekeeping gene comparator, suggesting that the treatments, at least in part, potentiate increases in neuronal frataxin.

Transcriptional repression of *FXN* is thought to result from reduced accessibility of transcriptional regulatory factors to the promoter region caused by the trinucleotide repeat expansion,^30^ and various regulatory factors binding close to the *FXN* gene locus are implicated^31^, ^32^, ^33^ (Fig 4A). Of these, we found that tumor protein p53 (p53; encoded by *Trp53*), transcription factor AP‐2 alpha (TFAP2A; encoded by *Tfap2a*), serum response factor (SRF; encoded by *Srf*), and hypoxia‐inducible factor‐2alpha (HIF‐2A; encoded by *Epas1*) were increased following cytokine administration (Fig 4C). Of the regulatory factors tested, only *Epas1* and *Trp53* were upregulated in both cerebellum and spinal cord in response to cytokine administration; and of these, only *Epas1* expression in treated mice correlated with respective *FXN* expression (Fig 4D).

We also showed marked increases in frataxin protein expression within the spinal cord, and to a lesser extent in the cerebellum, with G‐CSF or G‐CSF/SCF administration (using a human‐specific frataxin ELISA, as with qPCR analysis, did not allow comparisons between the “human” frataxin levels within YG8R mice and control mouse frataxin levels; Fig 4B).

Frataxin deficiency results in increased oxidative stress and impaired recruitment of antioxidant defences.^34^ In line with previous studies YG8R mice had reduced protein levels of PGC‐1a and Nrf2 in either the spinal cord and/or cerebellum.^35^, ^36^ This was associated with global reductions in expression of antioxidant enzymes and enzymatic activity of the iron‐sulphur protein aconitase within the cerebellum^28^ (Table). Untreated YG8R mice also showed increases in the lipid peroxidation product, 4‐HNE, within the cerebellum and spinal cord (see Table).

**Table 1:** Friedreich's Ataxia–Associated Molecules and Antioxidant Defenses Are Restored in Both the Cerebellum and Spinal Cord of YG8R Mice Treated With G‐CSF and/or SCF

Protein	Site	Level Relative to	Control	YG8R	YG8R+G‐CSF	YG8R+SCF	YG8R+G‐CSF/SCF
Friedreich's ataxia–associated molecules							
Nrf2	Cerebellum	Total protein	1.40 (0.08)^**^	1.00 (0.05)	1.12 (0.09)	1.40 (0.18)^*^	1.45 (0.10)^*^
		NeuN	1.27 (0.02)^*^	1.00 (0.09)	1.66 (0.17)^**^	1.54 (0.13)^*^	1.50 (0.11)^*^
	Spinal cord	Total protein	0.95 (0.05)	1.00 (0.07)	0.85 (0.10)	1.19 (0.11)	1.08 (0.11)
		NeuN	1.61 (0.26)	1.00 (0.16)	2.06 (0.26)^*^	1.88 (0.33)	2.06 (0.16)^*^
PGC‐1a	Cerebellum	Total protein	1.45 (0.08)^***^	1.00 (0.04)	1.23 (0.10)	1.31 (0.10)	1.38 (0.09)^*^
		NeuN	1.30 (0.02)^*^	1.00 (0.08)	1.81 (0.18)^**^	1.45 (0.13)	1.43 (0.14)
	Spinal cord	Total protein	1.18 (0.04)^*^	1.00 (0.06)	1.06 (0.07)	1.38 (0.09)^**^	1.20 (0.08)
		NeuN	1.96 (0.40)^*^	1.00 (0.20)	2.46 (0.25)^**^	2.09 (0.30)^*^	2.26 (0.26)^**^
Aconitase	Cerebellum	Total protein	1.76 (0.12)^***^	1.00 (0.06)	1.22 (0.16)	1.11 (0.10)	1.61 (0.10)^**^
		NeuN	1.54 (0.16)^**^	1.00 (0.07)	1.72 (0.02)^**^	1.24 (0.11)	1.70 (0.12)^**^
	Spinal cord	Total protein	1.03 (0.11)	1.00 (0.10)	1.79 (0.38)	0.94 (0.20)	1.83 (0.14)
		NeuN	1.70 (0.35)	1.00 (0.27)	4.07 (0.87)^**^	1.29 (0.23)	3.36 (0.34)^*^
Antioxidant enzyme/oxidative damage expression							
SOD1	Cerebellum	Total protein	1.37 (0.05)^**^	1.00 (0.05)	1.17 (0.08)	1.28 (0.07)^*^	1.22 (0.08)
		NeuN	1.22 (0.03)^*^	1.00 (0.06)	1.74 (0.15)^**^	1.44 (0.15)	1.27 (0.10)
	Spinal cord	Total protein	1.42 (0.11)^*^	1.00 (0.06)	1.01 (0.11)	1.54 (0.06)^**^	1.40 (0.18)
		NeuN	2.28 (0.31)^**^	1.00 (0.20)	2.30 (0.18)^**^	2.32 (0.31)^**^	2.60 (0.34)^**^
SOD2	Cerebellum	Total protein	1.30 (0.12)	1.00 (0.08)	1.34 (0.07)	1.72 (0.13)^***^	1.32 (0.14)
		NeuN	1.19 (0.11)	1.00 (0.07)	2.01 (0.22)^**^	1.97 (0.28)^**^	1.37 (0.13)
	Spinal cord	Total protein	1.39 (0.24)	1.00 (0.05)	2.21 (0.14)^***^	2.76 (0.26)^***^	1.79 (0.20)^*^
		NeuN	2.07 (0.25)	1.00 (0.23)	4.94 (0.43)^**^	3.96 (0.46)^*^	3.67 (1.16)^*^
Catalase	Cerebellum	Total protein	1.57 (0.13)^**^	1.00 (0.05)	1.23 (0.06)	1.42 (0.06)^**^	1.31 (0.12)^*^
		NeuN	1.45 (0.08)^**^	1.00 (0.05)	1.84 (0.19)^**^	1.63 (0.21)^*^	1.35 (0.10)
	Spinal cord	Total protein	1.40 (0.19)^*^	1.00 (0.06)	1.75 (0.12)^***^	2.04 (0.13)^***^	1.62 (0.12)^**^
		NeuN	1.84 (0.06)^*^	1.00 (0.23)	3.96 (0.47)^***^	2.95 (0.37)^*^	3.09 (0.64)^*^
GPX1	Cerebellum	Total protein	1.30 (0.08)^*^	1.00 (0.07)	1.21 (0.11)	1.40 (0.05)*	1.39 (0.12)^*^
		NeuN	1.51 (0.32)	1.00 (0.06)	1.78 (0.10)^***^	1.59 (0.12)^***^	1.44 (0.08)^*^
	Spinal cord	Total protein	0.93 (0.05)	1.00 (0.10)	0.91 (0.03)	1.25 (0.04)	1.30 (0.15)
		NeuN	1.69 (0.29)	1.00 (0.13)	2.29 (0.25)^**^	2.02 (0.23)^*^	2.06 (0.26)^*^
4‐HNE	Cerebellum	Total protein	0.55 (0.02)^*^	1.00 (0.13)	0.74 (0.06)	0.95 (0.02)	0.67 (0.09)^*^
		NeuN	n/a	n/a	n/a	n/a	n/a
	Spinal cord	Total protein	0.54 (0.08)^*^	1.00 (0.11)	0.88 (0.10)	0.85 (0.12)	0.67 (0.06)
		NeuN	n/a	n/a	n/a	n/a	n/a

The relative protein expression levels of frataxin; aconitase enzyme activity; transcription factors Nrf2 and PGC‐1a; antioxidant enzymes SOD1, SOD2, catalase, and GPX1, and lipid peroxidation product 4‐HNE, within the cerebellum and spinal cord of both WT controls and YG8R mice and YG8R mice treated with G‐CSF and/or SCF. All statistical comparisons are versus YG8R mice. Comparisons between control and untreated YG8R mice were analyzed using unpaired *t* tests or Mann–Whitney *U* tests. For all other analyses, either one‐way analysis of variance followed by Dunnett's multiple comparison test or Kruskal‐Wallis followed by Dunn's multiple comparison test was applied. *p < 0.05; **p < 0.01; ***p < 0.001. Values represent the mean ± standard error of the mean, relative to values in untreated YG8R mice. For all tests, n = 5 per genotype.

4‐HNE = 4‐hydroxynonenal; G‐CSF = granulocyte colony‐stimulating factor; GPX1 = glutathione peroxidase 1; n/a = not applicable; NeuN = neuronal nuclear antigen; Nrf2 = nuclear factor E2‐related factor 2; PGC‐1a = peroxisome proliferator‐activated receptor‐gamma coactivator 1 alpha; SCF = stem cell factor; SOD1/2 = superoxide dismutase 1 and 2; WT = wild type.

Administration of cytokines led to restoration of aconitase activity in cerebellum and spinal cord to, or beyond, levels observed in WT control mice. Similarly, there were increases in levels of PGC‐1a and Nrf2 protein expression with associated elevations in SOD1 and SOD2, catalase, and GPX1. Finally, consistent with cytokines facilitating redox homeostasis, in cerebellum and spinal cord, reductions in 4‐HNE were also apparent with combined administration of G‐CSF and SCF (see Table).

### G‐CSF and SCF Improve Nerve Conduction

**Figure 5:**
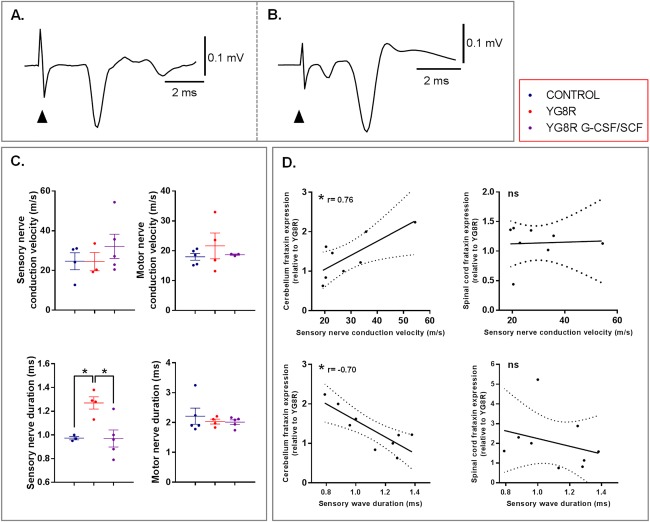
Neurophysiological deficits of the sensory nerve pathway are restored in YG8R mice treated with a combination of G‐CSF and SCF. (A) Sensory compound nerve recording from the proximal tail after stimulation at the tail tip. (B) Motor compound nerve recording from the distal tail after stimulation of the proximal tail. The first small negative wave is attributed to antidromic activation of sensory fibers. (A and B) Responses are an average of 10 trials and from control animals. Arrowhead indicates onset of the electrical stimulation. (C) Peak conduction velocities and durations of sensory and motor responses recorded from tails of wild‐type controls, untreated YG8R mice, and YG8R mice treated with combined G‐CSF/SCF. (D) Correlation and linear regression analysis of frataxin protein levels (normalized to NeuN) in the spinal cord and cerebellum of for untreated and treated YG8R mice with either sensory nerve conduction velocity or wave duration (lines of best fit and 95% confidence interval are depicted). All statistical comparisons are versus YG8R mice. One‐way analysis of variance, followed by Dunnett's multiple comparison test, was applied for all analyses. *p < 0.05; values represent means ± standard error of the mean. Spearman's correlation was used to analyze relationships between frataxin and sensory nerve conduction velocity or wave duration. r = correlation coefficient. G‐CSF = granulocyte colony‐stimulating factor; NeuN = neuronal nuclear antigen; ns = not significant; SCF = stem cell factor.

Given that Friedreich's ataxia patients exhibit abnormalities in nerve conduction,^37^ neurophysiological evaluation of sensory and motor nerve conduction was performed. Electrophysiological recordings were obtained from the tail nerves of WT controls, untreated YG8R mice, and YG8R mice treated with combined G‐CSF/SCF (Fig 5A–D). There was no statistical difference in the sensory or motor conduction velocities among all three groups regardless of whether the peak (Fig 5C) or onset latency was used. However, duration of the sensory compound nerve potential in untreated YG8R mice was significantly longer than that of WT controls, signifying temporal dispersion of the nerve impulses evoked by the stimulation. Furthermore, G‐CSF/SCF treatment restored nerve volley duration to normal levels (Fig 5C).

Electrophysiological abnormalities in Friedreich's ataxia correlate with GAA triplet repeat expansion length^38^ (and therefore reduced frataxin protein expression). We found that increases in cerebellar frataxin protein levels in G‐CSF/SCF‐treated mice significantly correlated with shorter duration and increased conduction velocity of sensory compound nerve potentials (Fig 5D). These findings provide support for a link between increases in frataxin and neurophysiological improvements observed in treated YG8R mice.

### G‐CSF and SCF Reduce DRG, Spinal Cord, and Cerebellar Friedreich's Ataxia–Related Pathology

**Figure 6:**
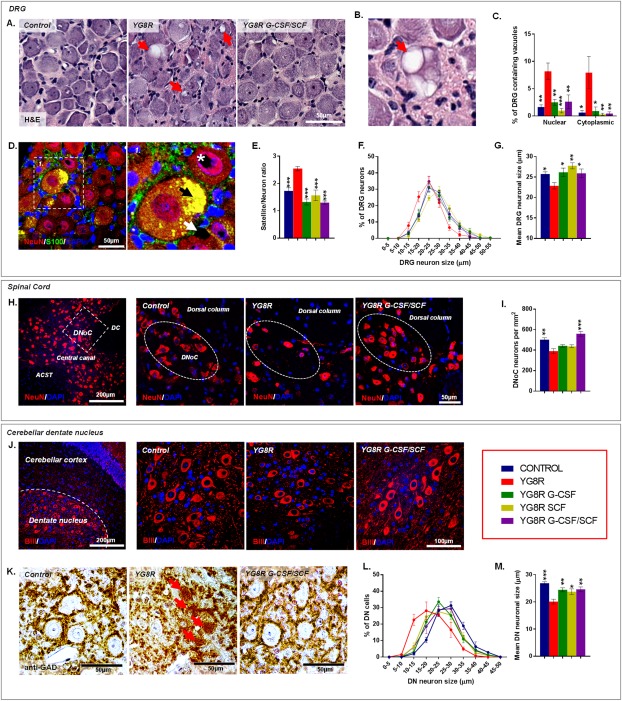
G‐CSF and SCF administration improves Friedreich's ataxia–associated pathology. (A) Hematoxylin and eosin–stained DRG depicting reductions in vacuolization (red arrows) of large sensory neurons within YG8R mice treated with G‐CSF/SCF. (B) High‐powered image of a DRG neuron showing significant vacuolisation (red arrow). (C) Frequency of DRG neurons containing nuclear or cytoplasmic vacuoles. (D) DRG sections labeled with NeuN and S100 showing autofluorescent lipofuscin (black arrow) and both intranuclear (white asterisk) and intracytoplasmic (white arrow) vacuolization. (E) DRG satellite‐to‐neuron cell ratio, (F) size range, and (G) mean cell size (diameter) of DRG neurons. (H) Images and (I) numbers of NeuN‐labeled neurons within the DNoC of YG8R mice treated with G‐CSF/SCF. (J) Images of beta‐3 tubulin‐expressing neurons and (K) grumose‐type GAD‐positive intracytoplasmic labeling pattern in and around the large neuronal cell bodies within the cerebellar dentate nucleus of control and YG8R mice. (L) Size range and (M) mean cell size (diameter) of beta‐3 tubulin‐labeled neurons within the cerebellar dentate nucleus. Comparisons between WT‐control and YG8R mice were compared using the unpaired *t* test. All other statistical comparisons are versus YG8R mice using either one‐way analysis of variance followed by Dunnett's multiple comparison test or Kruskal‐Wallis followed by Dunn's multiple comparison test. *p < 0.05; **p < 0.01; ***p < 0.001; values represent means ± standard error of the mean. For all tests, n = 5 per genotype. ACST = anterior corticospinal tract; BIII = beta‐3 tubulin; DAPI = 4',6‐diamidino‐2‐phenylindole; DC = dorsal column; DN = cerebellar dentate nucleus; DNoC = dorsal nucleus of Clarke; DRG = dorsal root ganglia; GAD = glutamate decarboxylase; G‐CSF = granulocyte colony‐stimulating factor; H&E = hematoxylin and eosin; NeuN = neuronal nuclear antigen; SCF = stem cell factor.

**Figure 7:**
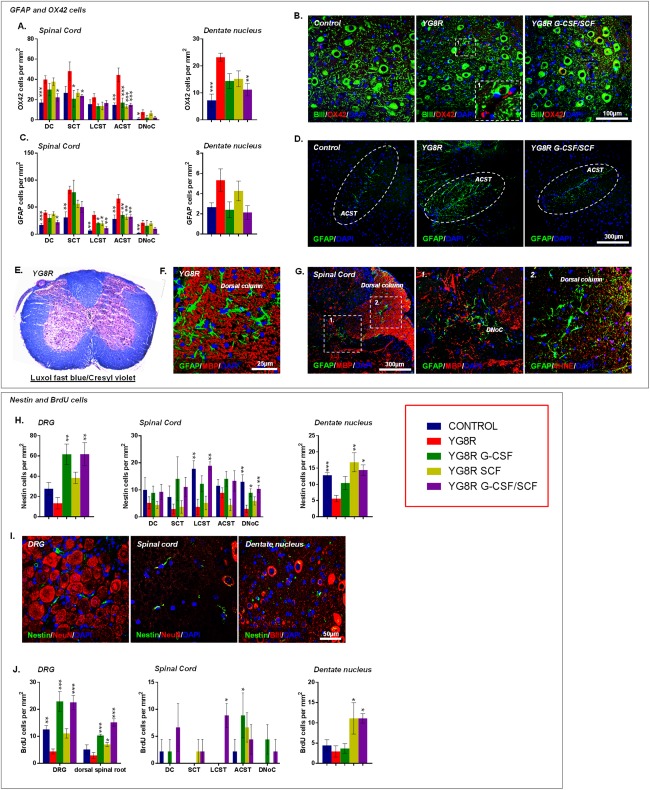
G‐CSF and SCF administration reduces glial/immune cell infiltration while stimulating the recruitment of neural precursors to areas of tissue injury. Numbers of (A) OX42‐ and (C) GFAP‐positive cells within the spinal cord and cerebellar dentate nucleus. (B) Cerebellar sections depicting levels of OX42‐positive cells in the cerebellar dentate nucleus. (D) Spinal cord sections depicting levels of GFAP‐positive cells within the spinal cord anterior corticospinal tract. Astrocytosis without loss of spinal cord white matter in YG8R mice observed using (E) Luxol fast blue/cresyl violet staining and (F) MBP‐dual immunolabeling with GFAP. (G) Spinal cord sections immunolabeled with GFAP and either MBP or 4‐HNE, exhibiting astrocytosis, in both the white and gray matter, associated with 4‐HNE accumulation. (H) Nestin cells/mm^2^ and (I) images of nestin‐positive cells within DRG, spinal cord, and cerebellar dentate nucleus. (J) BrdU cells/mm^2^ within DRG, spinal cord, and cerebellar dentate nucleus. Dorsal column (DC), spinocerebellar tract (SCT), lateral corticospinal tract (LCST), anterior corticospinal tract (ACST), and dorsal nucleus of Clarke (DNoC). Comparisons between WT‐control and YG8R mice were compared using the unpaired *t* test. All other statistical comparisons are versus YG8R mice using either one‐way analysis of variance followed by Dunnett's multiple comparison test or Kruskal‐Wallis followed by Dunn's multiple comparison test. *p < 0.05; **p < 0.01; ***p < 0.001; values represent means ± standard error of the mean. For all tests, n = 5 per genotype. 4‐HNE = 4‐hydroxynonenal; BIII = beta‐3 tubulin; BrdU = bromodeoxyuridine; DAPI = 4′,6‐diamidino‐2‐phenylindole; DRG = dorsal root ganglia; G‐CSF = granulocyte colony‐stimulating factor; GFAP = glial fibrillary acidic protein; MBP = myelin basic protein; NeuN = neuronal nuclear antigen; SCF = stem cell factor; WT = wild type.

We further characterized neuropathological changes in YG8R mice. In common with human Friedreich's ataxia^1^ and previous studies describing the YG8R mouse,^28^, ^39^ untreated YG8R mice displayed: frequent intra‐nuclear and ‐cytoplasmic vacuolization of the large sensory neuronal cell bodies of the DRG with significant lipofuscin accumulation (Fig 6A–D) and an increased satellite cell to DRG neuron ratio (Fig 6E) representing large sensory DRG neuronal loss (Fig 6F,G) and/or subsequent satellite cell proliferation; neuronal loss in the spinal cord dorsal nucleus of Clarke (DNoC; Fig 6H,I); atrophy of large neurons within the cerebellar dentate nucleus with grumose‐type GAD‐positive intracytoplasmic labeling pattern in the large neuronal cell bodies of the YG8R mice (Fig 6J–M); and spinal cord astrocytosis–associated 4‐HNE accumulation at the peripheral aspects of the anterior and lateral white matter tracts and extending into the dorsal columns (Fig 7C,D,G), although no significant white matter loss (Fig 7E,F). Patchy astrocytosis was also observed in the gray matter surrounding the central canal and extending toward the DNoC (Fig 7G); influx of inflammatory OX42 (CD11b/c)‐positive cells in both the spinal cord and cerebellar dentate nucleus (Fig 7A,B); and reduction in nestin‐positive cells in lateral corticospinal tracts, DNoC, and cerebellar dentate nucleus, compared to WT control mice (Fig 7H,I).

We observed clear attenuation of Friedreich's ataxia–associated pathology within the DRG, spinal cord, and cerebellum of YG8R mice treated with cytokines. All treatment regimens led to significant reductions in the number of DRG neurons containing either intracytoplasmic or ‐nuclear vacuoles (Fig 6A–C). Furthermore, treatment returned satellite‐cell‐to‐neuronal ratios to levels found in WT controls—a likely consequence of improved large sensory neuronal cell survival (Fig 6E–G). Combined G‐CSF/SCF treatment markedly reduced neuronal loss within the spinal cord DNoC (Fig 6H,I) and reduced the extent of astrocytosis and inflammatory cell infiltrate within dorsal columns, spinocerebellar, and corticospinal tracts (Fig 7A–D). In the cerebellar dentate nucleus, both G‐CSF and SCF reduced atrophy of large neurons (resulting in an increased mean neuronal cell size) and reduced the presence of grumose degeneration (Fig 6J–M).

To assess the effects of treatment on cellular kinetics, YG8R mice were injected with the thymidine analogue, BrdU, directly following cytokine administration during the final treatment round (Fig 1A). Histological analysis 1 month post‐BrdU exposure revealed a distinct reduction in BrdU positive cell numbers within DRG of untreated YG8R mice (compared to WT controls), but marked increases in BrdU positive cells in DRG, dorsal spinal roots, cerebellar dentate nucleus, and, to a lesser extent, in the spinal cord of G‐CSF‐ and/or SCF‐treated mice (Fig 7J). We also found that the pool of nestin‐positive neural precursor cells within the DRG, spinal cord, and cerebellar dentate nucleus was significantly amplified in response to treatment with G‐CSF and/or SCF (Fig 7H,I).

## Discussion

Here, we show that administration of G‐CSF and SCF have marked direct neuroprotective effects in a “humanized” mouse model of Friedreich's ataxia; these agents correct many Friedreich's ataxia–associated biochemical abnormalities and improve functional, neurophysiological, and pathological parameters. Significantly, administration of G‐CSF and/or SCF mediates sustained improvements in motor coordination and locomotor activity in YG8R “Friedreich's ataxia” mice, even after onset of clinical symptoms. Treatment also restored the duration of sensory nerve compound potentials, reflecting reduced variability in conduction velocities of individual nerve fibers associated with Friedreich's ataxia dysfunction.^37^, ^38^

G‐CSF and SCF had pronounced effects on frataxin levels and on regulators of frataxin expression within both the cerebellum and spinal cord. Of the potential transcription factors we tested,^31^, ^32^, ^33^ HIF‐2alpha encoded by *Epas1* was upregulated in the cerebellum and spinal cord in response to treatment and correlated with *FXN* expression, highlighting a possible regulatory mechanism by which G‐CSF and SCF control frataxin expression. Indeed, others have shown that HIF‐2alpha can activate the murine *FXN* promoter through binding to a consensus HIF‐responsive enhancer element, and mice lacking *Epas1* have markedly reduced levels of frataxin.^33^

Interventions that increase the amount of the frataxin protein are attractive therapeutic approaches. Carriers of the GAA expansion, having approximately 50% of normal frataxin expression, are asymptomatic.^5^, ^40^ Increasing cellular frataxin levels above a specific threshold therefore hold promise; a recent proof‐of‐concept study introducing *FXN* transgenes into heart cells of frataxin‐deficient mice led to overexpression of frataxin and sustained remission of Friedreich's ataxia–associated heart disease.^41^ Experimentally, several agents have shown potential to increase frataxin expression (recombinant erythropoietin, interferon‐gamma, nicotinamide, and resveratrol); however, there has been limited success in their capacity to elevate frataxin levels when tested clinically.^42^, ^43^, ^44^, ^45^

Molecules coupled with frataxin deficiency were also elevated in response to treatment. Specifically, G‐CSF and/or SCF increased expression of molecules associated with frataxin antioxidant functions, including Nrf2, SODs, catalase, and GPX1. Nrf2 is a key orchestrator of cellular antioxidant responses and its expression/activity is reduced in frataxin‐deficient cells, leading to increased oxidative injury.^35^, ^46^, ^47^ Cytokines also increased expression of PGC‐1a, another key regulator of cellular redox homeostasis,^48^ and reduced lipid peroxidation products,^49^ thus confirming their antioxidative effects.

YG8R mice replicate human disease in many histological aspects, with neuronal atrophy in the DRG, DNoC, and cerebellar dentate nucleus. Importantly, repeated administration of cytokines led to significant amelioration in disease‐related pathology throughout the nervous system, the likely explanation for the observed improvements in motor and locomotor function. Mechanistically, in addition to restoring frataxin‐associated cellular homeostasis, G‐CSF/SCF attenuating inflammation (both astro‐ and microgliosis) in the nervous system of YG8R mice may have also slowed the progression of the disease.^50^

Adult neurogenesis appears to be an important mechanism of brain plasticity in brain repair postinjury.^51^ We found reduced numbers of proliferating cells and neural precursors throughout the nervous system of untreated YG8R mice. This may be a consequence of mitochondrial dysfunction induced by frataxin deficiency,^52^ and abnormal neurogenesis may, in turn, exacerbate neuropathology.^53^ Both G‐CSF and SCF regulate proliferation, differentiation, and recruitment of endogenous neural and bone marrow progenitor cells during neurological injury.^17^, ^54^ In accord, cytokine administration increased the number of both nestin‐positive and proliferating cells in the YG8R nervous system. Of note, numbers of nestin cells were elevated in the DRG, DNoC, and cerebellar dentate nucleus, all areas in which neuronal preservation was apparent in response to treatment.

As demonstrated here, humanized mice are powerful tools in preclinical testing of potential therapeutic agents of neurological disease; however, care should be taken when interpreting data attributed to underlying genomic differences between rodents and humans. We believe these observations warrant further clinical trials of stem‐cell–mobilizing agents in patients with Friedreich's ataxia. When used clinically, both G‐CSF and SCF are generally well tolerated, with G‐CSF having a well‐established safety record in healthy peripheral blood stem cell donors. The pharmacokinetics of G‐CSF administration has been extensively studied in humans, and the therapeutic window, in terms of achieving HSC mobilization, can extend beyond twice the recommend dose.^55^ Furthermore, monthly administrations of mobilizing agents have been shown to be safe in a trial for amyotrophic lateral sclerosis.^19^ This has provided early safety data on the use of G‐CSF for neurodegenerative conditions.

In conclusion, these experiments have elucidated mechanisms of action of cytokines in a humanized Friedreich's ataxia mouse model. Their pleiotropic effects contribute to neuroprotection and repair against Friedreich's ataxia–associated pathological mechanisms, thereby offering a therapy that may reduce, or even help reverse, long‐term disability in patients with Friedreich's ataxia.

## Author Contributions

K.K., N.C., R.A., M.P., N.S., and A.W. were responsible for conception and design of the study. K.K., N.C., R.A., K.H., J.R., A.C., H.H., and B.B. were responsible for acquisition and analysis of data. K.K., A.W., N.S., N.C., and R.A. were responsible for drafting a significant proportion of the manuscript.

## Potential Conflicts of Interest

Nothing to report.
